# Selenium-Binding Protein 1 (SBP1): A New Putative Player of Stress Sensing in Plants

**DOI:** 10.3390/ijms25179372

**Published:** 2024-08-29

**Authors:** Irene Dervisi, Aikaterini Koletti, Adamantia Agalou, Kosmas Haralampidis, Emmanouil Flemetakis, Andreas Roussis

**Affiliations:** 1 Department of Botany, Faculty of Biology, National & Kapodistrian University of Athens, 15784 Athens, Greece; eidervisi@biol.uoa.gr (I.D.);; 2 Department of Biotechnology, School of Applied Biology and Biotechnology, Agricultural University of Athens, 11855 Athens, Greece; kolettik@aua.gr (A.K.); mflem@aua.gr (E.F.); 3 Laboratory of Toxicological Control of Pesticides, Scientific Directorate of Pesticides’ Control & Phytopharmacy, Benaki Phytopathological Institute (BPI), 14561 Athens, Greece; a.agalou@bpi.gr

**Keywords:** methanethiol oxidase, tolerance, oxidative stress, development, molecular networks

## Abstract

Selenium-binding proteins (SBPs) represent a ubiquitous and conserved protein family with yet unclear biochemical and molecular functions. The importance of the human homolog has been extensively studied as it is implicated in many cancer types and other diseases. On the other hand, little is known regarding plant homologs. In plants, there is evidence that SBP participates in developmental procedures, oxidative stress responses, selenium and cadmium binding, and pathogenic tolerance. Moreover, recent studies have revealed that SBP is a methanethiol oxidase (MTO) catalyzing the conversion of methanethiol into formaldehyde, H_2_S, and H_2_O_2_. The two later products emerge as key signal molecules, playing pivotal roles in physiological processes and environmental stress responses. In this review, we highlight the available information regarding plants in order to introduce and emphasize the importance of SBP1 and its role in plant growth, development, and abiotic/biotic stress.

## 1. The Discovery and Importance of Selenium in Plants

In 1817, Jöns Jakob Berzelius discovered selenium (Se), which was initially believed to be a toxic element for humans. However, in 1957, Klaus Schwartz and Calvin Foltz revealed the beneficial effects of selenium against liver necrosis. This discovery transformed our understanding of selenium and its potential health benefits [1].

While selenium is widely recognized as a beneficial element in plants, it is not classified as an essential element [2]. As a member of the metalloid group, also known as chalcogens, selenium is considered toxic and can be found in various environments across the globe [3]. Interestingly, selenium rarely occurs in its elemental form in nature [4]. Despite its toxic properties, selenium plays a vital role in the well-being of microbes, animals, and plants.

Selenium shares similarities with sulfur, another member of the chalcogen group, in terms of physical and chemical properties, oxidation states, and functional group types. However, distinct chemicals exist that explain why organisms utilize selenium instead of sulfur [5]. One notable difference is the increased polarizability of selenium, compared to sulfur, due to its heavier atomic weight. This higher polarizability leads to weaker selenium bonds and faster bond-breaking reactions. Consequently, selenium is more reactive as an electron acceptor compared to sulfur. Additionally, all oxidation states of selenium are significantly more electrophilic than their sulfur analogs. Furthermore, heavier elements like selenium exhibit greater tolerance for hypervalent bonding situations.

The significance of selenium for animals and microorganisms is exemplified by the presence of crucial enzymes called selenoproteins. These enzymes, such as thioredoxin reductases (TrxRs), glutathione peroxidase (GPxs), and iodothyronine deiodinases (DIOs), incorporate selenocysteine instead of cysteine [6]. Selenoproteins are encoded by a distinct codon, TGA, and their existence is well documented in various organisms, including algae. Interestingly, the presence of selenoproteins in other photosynthetic organisms has not been reported, despite the presence of organic and inorganic selenium [7].

In plants, selenium is incorporated into proteins through post-translation steps. This article aims to shed light on the protein family known as selenium-binding proteins (SBPs) in plants. Despite their name, SBPs are not members of the selenoprotein group. These highly conserved proteins have a wide distribution across all life kingdoms [8,9]. As their name suggests, SBPs are proteins that have been identified for their ability to bind selenium [10].

## 2. Discovery of SBP

Back in 1989, Bansal and his team made an intriguing finding while studying liver lysates from mice injected with ^75^Se—they stumbled upon a previously unknown protein weighing in at 56 kilodaltons [11]. This protein exhibited a unique ability to bind selenium, earning it the name selenium-binding protein 1 (SBP1) in later years [12].

When human SBP1 was successfully cloned, the resulting translated protein turned out to be 472 amino acids long [13]. What made SBP1 even more fascinating was its range of isoelectric points, hinting at possible modifications and further complexities [14,15].

The significance of SBP1 in human biology cannot be overstated. This remarkable protein has been linked to several cancer types and diseases, with its absence or reduced expression proving detrimental. For instance, thyroid cancer [16], lung cancer [17], stomach cancer [18,19], liver cancer [20,21], kidney cancer [22], ovarian cancer [23,24,25], breast cancer [26], prostate cancer [27,28,29], colon cancer [17,30,31], head and neck cancer [32], and malignant melanoma [33] have all been associated with SBP1 expression levels. The breadth of its impact on various cancer types underscores its importance in the field of oncology.

However, SBP1’s influence extends beyond cancer research. It has also captivated the attention of scientists exploring mental health disorders [34]. In fact, SBP1 has been proposed as a potential biomarker for schizophrenia [35,36,37], offering a glimmer of hope for improved diagnostics and treatment strategies in the realm of mental illness.

From its accidental discovery in mice to its pivotal role in human health, SBP1 continues to unfold, and significant efforts aim to uncover the intricate mechanisms underlying its functions, its potential as a therapeutic target, and the possibilities it holds for diagnostic advancements.

## 3. New Insights into the Role of SBPs

Researchers in 2017 identified a novel member of the selenium-binding protein family (SBP) known as MTO (methanethiol oxidase) in the bacterium Hypothmicrobium sp. VS [38]. This finding shed light on the previously unknown enzymatic activity of converting methanethiol to H_2_O_2_, formaldehyde, and H_2_S in humans, specifically through the human SBP homolog SELENBP1 [39].

Interestingly, mutations in SELENBP1 have been linked to extraoral halitosis, which is caused by increased levels of sulfur-containing metabolites in the body fluids and breath of affected individuals [39]. Methanethiol, a byproduct of bacterial metabolism, particularly in the gut [40], has been known to undergo oxidation. However, the specific enzyme responsible for this activity remained unidentified until now.

Furthermore, SELENBP1 has been found to interact with glutathione peroxidase 1 (GPX1), an antioxidant enzyme that converts peroxide to water, thereby mitigating its harmful effects [41]. These findings have led to the hypothesis that SELENBP1 plays a crucial role in sulfur metabolism and the production of H_2_O_2_ and H_2_S [39].

Building on these findings, Philipp and colleagues conducted experiments to clarify the function of SELENBP1 as a copper-dependent thiol oxidase, unaffected by selenium [42]. They proposed a copper-binding pocket consisting of the amino acids H^137^, H^140^, D^189^, and E^252^, which is essential for MTO activity. Furthermore, their study revealed that SELENBP1 can metabolize various thiols, with methanethiol and ethanethiol being the most promising substrates [42].

These new findings provide a deeper understanding of the role of SBPs in human physiology and metabolism. The identification of SELENBP1 as a methanethiol oxidase and its association with sulfur-related disorders and antioxidant enzymes offer potential avenues for further research and therapeutic interventions.

The recent biochemical discoveries surrounding SBPs, particularly SELENBP1, have unveiled novel enzymatic activities and their implications in sulfur metabolism and antioxidant defense mechanisms. These breakthroughs open up exciting possibilities for future studies and therapeutic advancements in various fields, including microbiology, biochemistry, and medicine.

## 4. Exploring the Role of SBPs in Plant Physiology

Since the discovery of selenium-binding proteins (SBPs) in plants, researchers have been uncovering the various roles and functions these proteins play in plant physiology. SBPs were first identified in *Physcomitrella patens* in 1999, where it was proposed that they might correlate with mammalian glutaredoxins (GPXs). This is due to the presence of a selenocysteine residue at the active site of GPXs, which acts as a co-factor. SBPs were suggested to sequester excess selenium in plants [43].

Further studies revealed that SBPs are involved in the plant’s defense against pathogens. In 2002, researchers treated rice plants with cerebroside elicitor from blast fungus and isolated five elicitor-responsive genes, one of which was *Os*SBP [44]. It was found that the expression of *Os*SBP, a rice homolog of SBP, increased in response to jasmonic acid and salicylic acid, hormones that are known for their role in stress responses, while abscisic acid and paraquat, compounds linked to the production of reactive oxygen species (ROS), caused alterations in *Os*SBP expression [45]. The overexpression of *Os*SBP was also found to enhance the plant’s tolerance to different pathogens, such as rice blast fungus (*Pyricularia grisea*) and rice bacterial blight (*Xanthomonas oryzae* pv. Oryzae) [46].

SBP homologs have been isolated and characterized in various plant species. In soybean, *Lotus japonicus*, and *Medicago sativa*, SBP homologs were found to have a role in controlling the oxidation/reduction status of target proteins, vesicular Golgi transport, and nodule formation and were initially characterized as nodulins [47,48,49]. Arabidopsis studies have shown that transgenic plants with higher expression of SBPs exhibit enhanced tolerance to selenite, while those with lower expression are more sensitive [50].

*Lj*SBP participates in a wide spectrum of physiological processes as it is expressed continuously at the phloem of *L. japonicus* root, siliques, and seedpods. Moreover, *Lj*SBP has a role in the formation and function of nodules during symbiotic procedures, through vesicle transportation from Golgi and ER for membrane synthesis [48]. The expression of *Lj*SBP increases in young nodules and other tissues with high energy requirements. Thus, *L. japonicus* SBP has been proposed as a crucial molecule for the procedures of organogenesis [48].

To further understand the interactions and functions of SBPs, researchers have performed yeast two-hybrid screenings and protein isolation experiments [8]. These studies have identified several proteins that interact with *At*SBPs, including a non-phosphorylating NADP-dependent glyceraldehyde-3-phosphate dehydrogenase activity (ALDH11A3), which was previously described as GAPDH, *At*FBA, glutaredoxins *At*GRXS14 and *At*GRXS16, phospholipase DAD1-like lipase 3, the protease *At*RD19c, and allergen *At*SAH7 [8,9,51,52,53,54], which are mentioned in the subsequent section.

SBPs have also been studied in other economically important plant species such as *Triticum aestivum* and *Theobroma cacao*. In *Triticum aestivum*, an SBP homolog called *Ta*SBP-A has been characterized as a protein that can alleviate photosynthesis impairment and oxidative damage caused by Cd stress [55]. The CXXC motif of *Ta*SBP-A plays a crucial role in Cd^2+^ binding, as the mutation of this motif led to a significant reduction in Cd binding [55]. Luo and colleagues reported *Ta*SBP-A as a cytosolic protein that is highly expressed in the root and plays a role in the detoxification of wheat from cadmium toxicity by diminishing its transfer of Cd from the root to the leaf [55]. Moreover, *Ta*SBP-A overexpression enhances plant growth, grain development, and selenium enrichment in wheat grains [56]. This increases the ability of the plant to bind and transport selenium in leaves and grains. Specifically, after the sodium selenite application, the selenium content within chloroplasts is enriched, and selenium is then transported through the phloem or neighboring internodes to the grains [56]. The study of Xiao and colleagues proposes *Ta*SBP-A as an appropriate candidate for selenium biofortification in wheat, conducing to the management of the worldwide issue of selenium deficiency in the human diet [56].

Furthermore, molecular docking studies predicted that the CXXC motif of SBPs in *Theobroma cacao* has an affinity for selenite [57]. More studies of *Tc*SBP revealed that it is a thermostable protein and is proposed to be involved in the late stages of witches’ broom disease, which is caused by the basidiomycete *Moniliophthora perniciosa* and destroys cacao cultures. From this perspective, *Tc*SBP is a suitable candidate for biotechnology approaches aimed at protecting cacao cultures from biotic as well as abiotic stress [57].

More recently, it has been discovered that SBP1 homologs in the model algae *Chlamydomonas reinhardtii*, known as *Cr*SBD1, act as stress sensors. This protein plays a vital role in early redox sensing and in triggering subsequent cellular responses as part of an extensive protein–protein interaction network. Interestingly, the *sbd1* mutant of *Chlamydomonas reinhardtii* appeared unaffected by a short-term H_2_O_2_ stress compared to the wild type, indicating an inability to perceive oxidative stress [58].

In conclusion, the study of SBPs in plants has revealed their diverse roles in plant physiology, including their involvement in selenium sequestration, defense against pathogens, and stress sensing. Further research is needed to fully understand the mechanisms through which SBPs function and their potential as targets for improving plant health and stress tolerance.

## 5. Structure

Selenium-binding protein (SBP) has captivated scientists with its elusive nature. Despite extensive research, the crystal structure of SBP has remained elusive, with only the X-ray structure of *Sulfolobus tokodaii* (PDB: 2ECE) serving as a reference point. This limited knowledge, however, has not deterred researchers from exploring the potential of SBP in various organisms, such as humans, Arabidopsis, and wheat, using 3D prediction and molecular docking based on the available structure.

Composed of a combination of α-helices and β-sheets, the structure of SBP takes on the form of a seven-blade propeller, enveloped by α-helices. Schild and colleagues have made significant strides in understanding the amino acids involved in selenium (Se) binding. Notably, in *At*SBP1, it has been predicted that Cys^21^Cys^22^ play a critical role in Se binding, while CSSC is essential for cadmium (Cd) binding. Interestingly, the Cys^5^XXCys^8^ motif has been proposed for Se binding in mammalian homologs, as the Cys^21^Cys^22^ motif is absent. Moreover, Cys^97^ and Cys^158^ form a disulfide bridge [59].

Certain motifs have been identified as conserved features among SBP proteins. These include CC/CXXC [59], KDEL, CSSC, HXD, and HXXHC [8,9,48,58]. The role of SBP in Se binding has been linked to the Cys^21^Cys^22^ motif in *Arabidopsis thaliana*, which is substituted with CxxC in most Chordata, suggesting its potential as a candidate for Se binding [59]. The KDEL motif, on the other hand, serves as an endoplasmic reticulum (ER) retention signal for many soluble proteins found in the cisternal lumen of eukaryotic cells [60]. The CSSC motif, which is found to be conserved in various species, is closely associated with redox activity [9,61,62,63,64,65]. The HXD motif, present in two parts of SBP, is believed to be a putative metal binding motif and plays a crucial role in redox signaling [66]. Additionally, the HXXHC motif has been implicated in substrate binding [67,68]. Two clathrin-binding boxes (pLφpφp) are also present, sequence traits linked to membrane trafficking [8,9]. All the conserved motifs of plant homologs are listed in Table 1.

Though SBP’s crystal structure remains an enigma, researchers continue to unravel its secrets through a combination of prediction techniques and molecular docking. As more is discovered about this mysterious protein, the potential for unlocking its applications in various organisms becomes increasingly tantalizing.

## 6. Exploring Gene Expression and Selenium Tolerance in Plants

In the realm of plant research, the focus on gene expression, promoter induction, and subcellular localization has primarily centered around Arabidopsis. However, there have been notable studies conducted on other crops such as wheat, rice, and cacao. Arabidopsis, in particular, harbors three alleles, referred to as *At*SBP1, *At*SBP2, and *At*SBP3.

Interestingly, *At*SBP1 and *At*SBP2 are arranged in a head-to-tail configuration on chromosome IV, while *At*SBP3 resides on chromosome III. The coding regions of AtSBP1 and AtSBP2 share 85% and 69% identity with *At*SBP3, respectively. Notably, prSBP1 is induced in the central cylinder of 10-day-old seedlings, whereas prSBP2 and prSBP3 exhibit induction in the same tissue as early as 2-day-old seedlings. Furthermore, prSBP3 is expressed in a distinct “fork”-like structure positioned just above the root meristem in the lower cells of the cortical cell files of both the central and lateral roots. prSBP1 and prSBP2 also display induction in the lateral roots, specifically in the columella initial cells. Moving on to cotyledons and leaves, all three promoters are activated in the vascular system, mesophyll cells, guard cells of the stomata, and hydathodes. During reproductive development, only prSBP1 and prSBP2 demonstrate activity, while prSBP3 is exclusively observed in the hydathodes of the cauline leaves [69].

To delve deeper into the effects of selenium compounds on the activation of all SBPs, Valassakis and colleagues conducted a study [69]. Surprisingly, no impact of selenium was observed on prSBP1, whereas prSBP2 and prSBP3 exhibited a robust response to sodium selenite compared to selenate in the root stele. Additionally, differences were noted in prSBP3 within cotyledons [69].

In terms of selenium tolerance, relative expression studies have shed light on the various patterns among the different *sbp* genes. *sbp1* shows ubiquitous expression, with the highest levels found in 3-day-old seedlings. *sbp2* is also widely expressed, with significant activity in leaves and shoots. On the other hand, *sbp3* is expressed in 3-day-old seedlings and roots of 10-day-old seedlings [69,70]. It is worth noting that *sbp1* is induced under cadmium treatment in roots [52,71]. Furthermore, *sbp1*, *sbp2*, and *sbp3* were downregulated in leaves after experiencing wounds [52].

When examining the subcellular level, all SBPs were found to be expressed in both the cytoplasm and the nucleus within the protoplast system [51]. However, in wheat, the homolog of these SBPs was solely localized in the cytoplasm [55].

These findings provide valuable insights into gene expression and selenium tolerance in plants. By delving into the intricate details of promoter induction, subcellular localization, and relative expression patterns, researchers are able to deepen their understanding of plant biology and potentially uncover strategies for enhancing plant resilience and tolerance to various environmental stressors.

## 7. Unveiling the Intricate Web of Protein Interactions in Plant Selenium Metabolism

In plant biology, the intricacies of protein networks continue to captivate researchers seeking to unravel the mysteries of cellular processes. One such network is the regulation of selenium metabolism in plants. Spearheading this exploration is the work of Agalou and colleagues, who proposed *At*SBP1’s involvement in a novel protein network.

Through investigations employing the yeast two-hybrid system and pull-down assays, Agalou et al. confirmed the interactions between *At*SBP1 and two other key players: a non-phosphorylating NADP-dependent glyceraldehyde-3-phosphate dehydrogenase activity (ALDH11A3), which was previously described as GAPDH and FBA. Yeast two-hybrid screening unveiled *At*SBP1’s ability to engage in a complex dance with 14 more proteins associated with vesicle trafficking, membrane synthesis, and oxidation/reduction control [8].

Over the years, further evidence has emerged, solidifying the existence of these interactions. Notably, the interactions between *At*SBP1 and two glutaredoxins, *At*GRXS14 and *At*GRXS16, were confirmed using both the yeast two-hybrid system and BiFC in protoplasts [51]. GRXs are small and ubiquitous glutathione (GSH)- or thioredoxin reductase (TR)-dependent oxidoreductases belonging to the highly conserved thioredoxin (TRX) superfamily [72,73,74,75]. As members of the plastidial class II GRXs, *At*GRXS14 and *At*GRX16 also possess Fe-S ligase activity and contain the GRX PICOT-like domain [51,76]. Most of the proteins containing PICOT-HD harbor Trx-HD, like *At*GRXS16. It is known that proteins with Trx-HD are important in regulating cellular redox state [77]. As a crucial component of the oxidative stress response and physiological functions, *At*GRXs’ interaction with *At*SBP1 sheds light on the intricate mechanisms that plants employ to combat environmental challenges. Interestingly, despite their disparate subcellular locations (chloroplasts for *At*GRXs and nucleus/cytoplasm for *At*SBP1), the confirmed interaction occurs in the cytoplasm, highlighting the dynamic nature of these protein associations. Moreover, Valassakis and colleagues showed that *At*SBP1 interacts with the N-terminal region upstream of the PICOT domain; thus, the PICOT domain is not important for the interaction [51]. Considering the properties and the suggested functions of the participating proteins, it is plausible to speculate that this network is part of the plant’s response to oxidative stress. As described before, *At*SBP1 possesses the sequence motif CSSC, which is associated with redox activity. It is also important to mention that glutaredoxins transfer electrons from glutathione to Cys residues. Thus, glutaredoxins can recycle SBP1 to be reused for further protein oxidation.

Another intriguing interaction involves the phospholipase *At*DALL3, a lipase implicated in jasmonic acid production and responses to wounding and stress including chemical treatment with selenium compounds and cadmium [52]. *At*DALL3’s subcellular localization in speckle-like structures within chloroplasts aligns with the site of its interaction with *At*SBP1 [52]. Such disparities in subcellular compartmentalization further emphasize the complexity of these protein networks. It is known that heavy metals cause modifications in the lipid cellular component, a process dependent on phospholipase activity [78]. Furthermore, cadmium and ROS can lead to the mobilization of proteins including phospholipases C and D, producing secondary messenger molecules [79,80]. Interestingly, the oxidative stress caused by Cd and other heavy metals increases the production of jasmonic acid, affecting growth procedures [81,82,83,84,85]. The upregulation of *At*DALL3 after Cd treatment could lead to JA upregulation as this phospholipase participates in JA biosynthesis and induces responses via *At*SBP1 interaction. Additional studies exploring the response of *At*SBP1 after wounding revealed its downregulation, whereas *At*DALL3 was upregulated. However, it has been reported that other stress sensors are downregulated [86]. Thus, it is tempting to speculate that at least in response to wounding, the downregulation of SBP genes may be considered part of a stress signaling mechanism. Moreover, it is assumed that this interaction alters chloroplast composition and the production of jasmonic acid.

Moving further, *At*SBP1’s reach extends to the papain-like cysteine protease *At*RD19c. Known for its involvement in anther development and programmed cell death (PCD), *At*RD19c’s subcellular localization in the intramembrane system and vacuoles contrasts with its interaction with AtSBP1 in the cytosol [53,87,88]. This enigmatic interplay between proteins underscores the delicate balance required for proper cellular functioning. In addition, *At*RD19c may be a part of the redox response network caused by Se. Thus, it is proposed that *At*RD19c plays a role in the plant response to oxidative stress via the interaction with *At*SBP1, which leads to PCD [53]. Another hypothesis is that this interaction results in the proteolysis of other proteins aiming at the production of molecules that act as secondary signaling molecules.

Delving deeper into the intricate web of protein interactions, *At*SBP1 surprises once again by engaging with *At*SAH7, an Ole e 1 allergen whose function remains elusive. *At*SAH7’s horseshoe-like expression structure enveloping the nucleus and ER serves as the backdrop for its interaction with *At*SBP1 [54]. These unexpected associations underscore the interconnectedness of seemingly disparate elements within the cell. *At*SAH7 participates in regulating responses to selenite and probably plays a role in oxidative stress. The probable correlation of *At*SAH7 with ABA and ROS and the expression of *At*SBP1 in guard cells implies a role of *At*SBP1/*At*SAH7 interaction in stomatal closure under stress. A possible role of the *At*SBP1 and *At*SAH7 complex can be the promotion of antioxidant production and the activation of defense responses triggered by ER stress [54].

**Figure 1:**
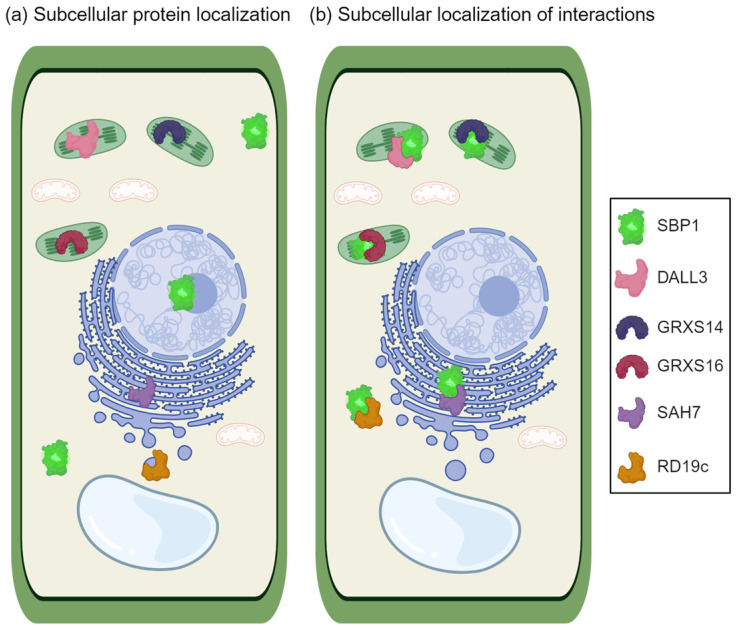
Schematic representation of subcellular localization of SBP1, DALL3, GRXS14, GRXS16, SAH7, and RD19c of *Arabidopsis thaliana* (**a**), and the subcellular localization of the protein interactions with SBP1 (**b**) (created with BioRender.com).

All aforementioned proteins are schematically represented in Figure 1, with respect to their subcellular localization and their site of interaction with SBP1. Expanding the scope of these findings, some of these interactions have also been confirmed in *Chamydomonas reinhardtii* using the yeast two-hybrid system [58]. This cross-validation across different plant species further solidifies the significance of these protein interactions.

The intricate web of protein interactions involving SBP1 presents new avenues for understanding cellular processes. These revelations not only shed light on the delicate balance within plant cells but also provide insights into potential strategies for optimizing selenium metabolism in agriculture and human health.

## 8. Proposed Biochemical/Molecular Action of SBP

In recent years, significant strides in understanding the role of selenium-binding proteins (SBPs) in various biological processes have been made. The discovery of SBP1 as a methanethiol oxidase has shed new light on its multifunctionality, presenting us with a fresh perspective on the intricate workings of this protein.

The proposed mechanism suggests that SBP1 catalyzes the conversion of methanethiol into formaldehyde, H_2_S, and H_2_O_2_. Among these byproducts, H_2_S and H_2_O_2_ emerge as key signal molecules, playing pivotal roles in physiological processes and environmental stress responses. H_2_S, in particular, has been implicated in moderating the effects of adverse conditions, activating enzymatic and non-enzymatic antioxidant systems to counteract oxidative damage through the post-translational modification (PTM) of proteins, specifically generating cysteine persulfides in specific residues.

To further support this hypothesis, researchers conducted a study on *Caenorhabditis elegans*, where they observed a reduction in H_2_S production and increased resistance to acute oxidative stress upon the knockout of SEMO-1, the SBP1 ortholog in the species [42,89]. These findings provide compelling evidence for SBP1’s involvement in H_2_S production and its potential impact on stress response.

Considering the above evidence, it is plausible to suggest that SBP1 may also serve as a conduit for H_2_S in plants, triggering the activation of various signal networks. This opens up exciting possibilities for exploring the intricate interplay between SBP1, H_2_S, and plant stress responses.

In conclusion, our evolving understanding of SBP1’s biochemical functions has revealed several possibilities and implications. The discovery of SBP1 as a methanethiol oxidase and its potential role in producing H_2_S and H_2_O_2_ signaling molecules represents a significant milestone in our effort to understand the function of this protein. Further research will undoubtedly unearth more insights into the complex mechanisms underlying SBP1’s functions and its impact on stress responses in diverse organisms.

## 9. Conclusions

SBPs possess diverse roles in plant physiology, such as selenium sequestration, defense against pathogens, and stress sensing. Moreover, SBPs are involved in selenium metabolism, providing insights into potential strategies for optimizing selenium metabolism in agriculture, phytoremediation, and biotechnological approaches for agriculture protection. SBPs participate in a protein stress sensing network providing further applications in agriculture aiming at the protection of crops from abiotic and biotic stress. The recent biochemical discoveries regarding novel enzymatic activities revealed their implications in sulfur metabolism and antioxidant defense mechanisms. These breakthroughs open up exciting possibilities for future studies and therapeutic advancements in various fields, including microbiology, biochemistry, and agriculture, as well as medicine.

## Figures and Tables

**Table 1:** Conserved motifs present in plant homologs. Positions are numbered based on *Arabidopsis thaliana* SBP1 (p is polar residue and φ is a bulky hydrophobic amino acid).

Conserved Motif	Position (aa)	Function
CC	21–22	Se binding
KDEL	86–89	Endoplasmic reticulum signal
CSSC	97–100	Redox activity
HxD	101–103, 347–349	Metal binding
HXXHC	154–158	Substrate binding
Clathrin-binding boxes (pLφpφp)	331–336, 460–465	Membrane trafficking

## References

[1] Schwarz K., Foltz C.M. (1957). Selenium as an Integral Part of Factor 3 against Dietary Necrotic Liver Degeneration. J. Am. Chem. Soc..

[2] Hasanuzzaman M., Bhuyan M.H.M.B., Raza A., Hawrylak-Nowak B., Matraszek-Gawron R., Mahmud J.A., Nahar K., Fujita M. (2020). Selenium in Plants: Boon or Bane?. Environ. Exp. Bot..

[3] Floor G.H., Román-Ross G. (2012). Selenium in Volcanic Environments: A Review. Appl. Geochem..

[4] Boyd R. (2011). Selenium Stories. Nat. Chem..

[5] Reich H.J., Hondal R.J. (2016). Why Nature Chose Selenium. ACS Chem. Biol..

[6] Lu J., Holmgren A. (2009). Selenoproteins. J. Biol. Chem..

[7] Bodnar M., Konieczka P., Namiesnik J. (2012). The Properties, Functions, and Use of Selenium Compounds in Living Organisms. J. Environ. Sci. Health Part C.

[8] Agalou A., Spaink H.P., Roussis A. (2006). Novel Interaction of Selenium-Binding Protein with Glyceraldehyde-3-Phosphate Dehydrogenase and Fructose-Bisphosphate Aldolase of Arabidopsis Thaliana. Funct. Plant Biol..

[9] Dervisi I., Valassakis C., Koletti A., Kouvelis V.N., Flemetakis E., Ouzounis C.A., Roussis A. (2023). Evolutionary Aspects of Selenium Binding Protein (SBP). J. Mol. Evol..

[10] Bansal M.P., Mukhopadhyay T., Scott J., Cook R.G., Mukhopadhyay R., Medina D. (1990). DNA Sequencing of a Mouse Liver Protein That Binds Selenium: Implications for Selenium’s Mechanism of Action in Cancer Prevention. Carcinogenesis.

[11] Bansal M.P., Cook R.G., Danielson K.G., Medina D. (1989). A 14-Kilodalton Selenium-Binding Protein in Mouse Liver Is Fatty Acid-Binding Protein. J. Biol. Chem..

[12] Giometti C.S., Liang X., Tollaksen S.L., Wall D.B., Lubman D.M., Subbarao V., Sambasiva Rao M. (2000). Mouse Liver Selenium-Binding Protein Decreased in Abundance by Peroxisome Proliferators. Electrophoresis.

[13] Chang P.W.G., Tsui S.K.W., Liew C., Lee C., Waye M.M.Y., Fung K. (1997). Isolation, Characterization, and Chromosomal Mapping of a Novel cDNA Clone Encoding Human Selenium Binding Protein. J. Cell. Biochem..

[14] Torrealba J.R., Colburn M., Golner S., Chang Z., Scheunemann T., Fechner J.H., Roenneburg D., Hu H., Alam T., Kim H.T. (2005). Selenium-Binding Protein-1 in Smooth Muscle Cells Is Downregulated in a Rhesus Monkey Model of Chronic Allograft Nephropathy. Am. J. Transplant..

[15] Chen G., Wang H., Miller C.T., Thomas D.G., Gharib T.G., Misek D.E., Giordano T.J., Orringer M.B., Hanash S.M., Beer D.G. (2004). Reduced Selenium-binding Protein 1 Expression Is Associated with Poor Outcome in Lung Adenocarcinomas. J. Pathol..

[16] Brown L.M., Helmke S.M., Hunsucker S.W., Netea-Maier R.T., Chiang S.A., Heinz D.E., Shroyer K.R., Duncan M.W., Haugen B.R. (2006). Quantitative and Qualitative Differences in Protein Expression between Papillary Thyroid Carcinoma and Normal Thyroid Tissue. Mol. Carcinog..

[17] Zhu Y., Pu Q., Zhang Q., Liu Y., Ma Y., Yuan Y., Liu L., Zhu W. (2023). Selenium-Binding Protein 1 Inhibits Malignant Progression and Induces Apoptosis via Distinct Mechanisms in Non-Small Cell Lung Cancer. Cancer Med..

[18] Zhang C., Xu W., Pan W., Wang N., Li G., Fan X., Xu X., Shen S., Das U.N. (2013). Selenium-Binding Protein 1 May Decrease Gastric Cellular Proliferation and Migration. Int. J. Oncol..

[19] Xia Y.-J., Ma Y.-Y., He X.-J., Wang H.-J., Ye Z.-Y., Tao H.-Q. (2011). Suppression of Selenium-Binding Protein 1 in Gastric Cancer Is Associated with Poor Survival. Hum. Pathol..

[20] Gao P.-T., Ding G.-Y., Yang X., Dong R.-Z., Hu B., Zhu X.-D., Cai J.-B., Ji Y., Shi G.-M., Shen Y.-H. (2018). Invasive Potential of Hepatocellular Carcinoma Is Enhanced by Loss of Selenium-Binding Protein 1 and Subsequent Upregulation of CXCR4. Am. J. Cancer Res..

[21] Huang C., Ding G., Gu C., Zhou J., Kuang M., Ji Y., He Y., Kondo T., Fan J. (2012). Decreased Selenium-Binding Protein 1 Enhances Glutathione Peroxidase 1 Activity and Downregulates HIF-1α to Promote Hepatocellular Carcinoma Invasiveness. Clin. Cancer Res..

[22] Ha Y.-S., Lee G.T., Kim Y.-H., Kwon S.Y., Choi S.H., Kim T.-H., Kwon T.G., Yun S.J., Kim I.Y., Kim W.-J. (2014). Decreased Selenium-Binding Protein 1 mRNA Expression Is Associated with Poor Prognosis in Renal Cell Carcinoma. World J. Surg. Oncol..

[23] Huang K., Park D.C., Ng S., Lee J.Y., Ni X., Ng W., Bandera C.A., Welch W.R., Berkowitz R.S., Mok S.C. (2006). Selenium Binding Protein 1 in Ovarian Cancer. Int. J. Cancer.

[24] Zhang C., Wang Y.E., Zhang P., Liu F., Sung C.J., Steinhoff M.M., Quddus M.R., Lawrence W.D. (2010). Progressive Loss of Selenium-Binding Protein 1 Expression Correlates with Increasing Epithelial Proliferation and Papillary Complexity in Ovarian Serous Borderline Tumor and Low-Grade Serous Carcinoma. Hum. Pathol..

[25] Yu-Rice Y., Edassery S.L., Urban N., Hellstrom I., Hellstrom K.E., Deng Y., Li Y., Luborsky J.L. (2017). Selenium-Binding Protein 1 (SBP1) Autoantibodies in Ovarian Disorders and Ovarian Cancer. Reproduction.

[26] Zhang S., Li F., Younes M., Liu H., Chen C., Yao Q. (2013). Reduced Selenium-Binding Protein 1 in Breast Cancer Correlates with Poor Survival and Resistance to the Anti-Proliferative Effects of Selenium. PLoS ONE.

[27] Ansong E., Ying Q., Ekoue D.N., Deaton R., Hall A.R., Kajdacsy-Balla A., Yang W., Gann P.H., Diamond A.M. (2015). Evidence That Selenium Binding Protein 1 Is a Tumor Suppressor in Prostate Cancer. PLoS ONE.

[28] Jeong J.-Y., Zhou J.-R., Gao C., Feldman L., Sytkowski A.J. (2014). Human Selenium Binding Protein-1 (hSP56) Is a Negative Regulator of HIF-1α and Suppresses the Malignant Characteristics of Prostate Cancer Cells. BMB Rep..

[29] Elhodaky M., Hong L.K., Kadkol S., Diamond A.M. (2020). Selenium-Binding Protein 1 Alters Energy Metabolism in Prostate Cancer Cells. Prostate.

[30] Wang N., Chen Y., Yang X., Jiang Y. (2014). Selenium-Binding Protein 1 Is Associated with the Degree of Colorectal Cancer Differentiation and Is Regulated by Histone Modification. Oncol. Rep..

[31] Zhang X., Hong R., Bei L., Yang J., Zhao X., Hu Z., Chen L., Meng H., Zhang Q., Niu G. (2022). Selenium Binding Protein 1 Inhibits Tumor Angiogenesis in Colorectal Cancers by Blocking the Delta-like Ligand 4/Notch1 Signaling Pathway. Transl. Oncol..

[32] Chen F., Chen C., Qu Y., Xiang H., Ai Q., Yang F., Tan X., Zhou Y., Jiang G., Zhang Z. (2016). Selenium-Binding Protein 1 in Head and Neck Cancer Is Low-Expression and Associates with the Prognosis of Nasopharyngeal Carcinoma. Medicine.

[33] Schott M., de Jel M.M., Engelmann J.C., Renner P., Geissler E.K., Bosserhoff A.K., Kuphal S. (2018). Selenium-Binding Protein 1 Is down-Regulated in Malignant Melanoma. Oncotarget.

[34] Jia Y., Zhang X., Wang Y., Liu Y., Dai J., Zhang L., Wu X., Zhang J., Xiang H., Yang Y. (2024). Knocking out Selenium Binding Protein 1 Induces Depressive-Like Behavior in Mice. Biol. Trace Elem. Res..

[35] Glatt S.J., Everall I.P., Kremen W.S., Corbeil J., Šášik R., Khanlou N., Han M., Liew C.-C., Tsuang M.T. (2005). Comparative Gene Expression Analysis of Blood and Brain Provides Concurrent Validation of *SELENBP1* up-Regulation in Schizophrenia. Proc. Natl. Acad. Sci. USA.

[36] Kanazawa T., Chana G., Glatt S.J., Mizuno H., Masliah E., Yoneda H., Tsuang M.T., Everall I.P. (2008). The Utility of SELENBP1 Gene Expression as a Biomarker for Major Psychotic Disorders: Replication in Schizophrenia and Extension to Bipolar Disorder with Psychosis. Am. J. Med. Genet. B Neuropsychiatr. Genet..

[37] Chau E.J., Mostaid M.S., Cropley V., McGorry P., Pantelis C., Bousman C.A., Everall I.P. (2018). Downregulation of Plasma SELENBP1 Protein in Patients with Recent-Onset Schizophrenia. Prog. Neuropsychopharmacol. Biol. Psychiatry.

[38] Eyice Ö., Myronova N., Pol A., Carrión O., Todd J.D., Smith T.J., Gurman S.J., Cuthbertson A., Mazard S., Mennink-Kersten M.A. (2018). Bacterial SBP56 Identified as a Cu-Dependent Methanethiol Oxidase Widely Distributed in the Biosphere. ISME J..

[39] Pol A., Renkema G.H., Tangerman A., Winkel E.G., Engelke U.F., De Brouwer A.P.M., Lloyd K.C., Araiza R.S., Van Den Heuvel L., Omran H. (2018). Mutations in SELENBP1, Encoding a Novel Human Methanethiol Oxidase, Cause Extraoral Halitosis. Nat. Genet..

[40] Blom H.J., Tangerman A. (1988). Methanethiol Metabolism in Whole Blood. J. Lab. Clin. Med..

[41] Fang W., Goldberg M.L., Pohl N.M., Bi X., Tong C., Xiong B., Koh T.J., Diamond A.M., Yang W. (2010). Functional and Physical Interaction between the Selenium-Binding Protein 1 (SBP1) and the Glutathione Peroxidase 1 Selenoprotein. Carcinogenesis.

[42] Philipp T.M., Gernoth L., Will A., Schwarz M., Ohse V.A., Kipp A.P., Steinbrenner H., Klotz L.-O. (2023). Selenium-Binding Protein 1 (SELENBP1) Is a Copper-Dependent Thiol Oxidase. Redox Biol..

[43] Machuka J., Bashiardes S., Ruben E., Spooner K., Cuming A., Knight C., Cove D. (1999). Sequence Analysis of Expressed Sequence Tags from an ABA-Treated cDNA Library Identifies Stress Response Genes in the Moss Physcomitrella Patens. Plant Cell Physiol..

[44] Sawada K., Iwata M. (2002). Isolation of Blast Fungal Cerebroside Elicitor-Responsive Genes in Rice Plants. J. Gen. Plant Pathol..

[45] Sawada K., Tokuda L., Shinmyo A. (2003). Characterization of the Rice Blast Fungal Elicitor Responsive Gene OSSBP Encoding a Homolog to the Mammalian Selenium—Binding Protelns. Plant Biotechnol..

[46] Sawada K., Hasegawa M., Tokuda L., Kameyama J., Kodama O., Kohchi T., Yoshida K., Shinmyo A. (2004). Enhanced Resistance to Blast Fungus and Bacterial Blight in Transgenic Rice Constitutively Expressing *OsSBP*, a Rice Homologue of Mammalian Selenium-Binding Proteins. Biosci. Biotechnol. Biochem..

[47] Gloudemans T., Bisseling T. (1989). Plant Gene Expression in Early Stages of Rhizobium-Legume Symbiosis. Plant Sci..

[48] Flemetakis E., Agalou A., Kavroulakis N., Dimou M., Martsikovskaya A., Slater A., Spaink H.P., Roussis A., Katinakis P. (2002). *Lotus japonicus* Gene *Ljsbp* Is Highly Conserved Among Plants and Animals and Encodes a Homologue to the Mammalian Selenium-Binding Proteins. Mol. Plant-Microbe Interact..

[49] Oehrle N.W., Sarma A.D., Waters J.K., Emerich D.W. (2008). Proteomic Analysis of Soybean Nodule Cytosol. Phytochemistry.

[50] Agalou A., Roussis A., Spaink H.P. (2005). The Arabidopsis Selenium-Binding Protein Confers Tolerance to Toxic Levels of Selenium. Funct. Plant Biol..

[51] Valassakis C., Dervisi I., Agalou A., Papandreou N., Kapetsis G., Podia V., Haralampidis K., Iconomidou V.A., Spaink H.P., Roussis A. (2019). Novel Interactions of Selenium Binding Protein Family with the PICOT Containing Proteins AtGRXS14 and AtGRXS16 in Arabidopsis Thaliana. Plant Sci..

[52] Dervisi I., Valassakis C., Agalou A., Papandreou N., Podia V., Haralampidis K., Iconomidou V.A., Kouvelis V.N., Spaink H.P., Roussis A. (2020). Investigation of the Interaction of DAD1-LIKE LIPASE 3 (DALL3) with Selenium Binding Protein 1 (SBP1) in Arabidopsis Thaliana. Plant Sci..

[53] Dervisi I., Haralampidis K., Roussis A. (2022). Investigation of the Interaction of a Papain-like Cysteine Protease (RD19c) with Selenium-Binding Protein 1 (SBP1) in Arabidopsis Thaliana. Plant Sci..

[54] Dervisi I., Petropoulos O., Agalou A., Podia V., Papandreou N., Iconomidou V.A., Haralampidis K., Roussis A. (2023). The SAH7 Homologue of the Allergen Ole e 1 Interacts with the Putative Stress Sensor SBP1 (Selenium-Binding Protein 1) in Arabidopsis Thaliana. Int. J. Mol. Sci..

[55] Luo F., Zhu D., Sun H., Zou R., Duan W., Liu J., Yan Y. (2023). Wheat Selenium-Binding Protein TaSBP-A Enhances Cadmium Tolerance by Decreasing Free Cd^2+^ and Alleviating the Oxidative Damage and Photosynthesis Impairment. Front. Plant Sci..

[56] Xiao T., Qiang J., Sun H., Luo F., Li X., Yan Y. (2024). Overexpression of Wheat Selenium-Binding Protein Gene TaSBP-A Enhances Plant Growth and Grain Selenium Accumulation under Spraying Sodium Selenite. Int. J. Mol. Sci..

[57] Martins Alves A.M., Pereira Menezes S., Matos Lima E., Peres Gramacho K., Silva Andrade B., Macêdo Ferreira M., Pirovani C.P., Micheli F. (2019). The Selenium-Binding Protein of Theobroma Cacao: A Thermostable Protein Involved in the Witches’ Broom Disease Resistance. Plant Physiol. Biochem..

[58] Koletti A., Dervisi I., Kalloniati C., Zografaki M.-E., Rennenberg H., Roussis A., Flemetakis E. (2022). Selenium-Binding Protein 1 (SBD1): A Stress Response Regulator in *Chlamydomonas reinhardtii*. Plant Physiol..

[59] Schild F., Kieffer-Jaquinod S., Palencia A., Cobessi D., Sarret G., Zubieta C., Jourdain A., Dumas R., Forge V., Testemale D. (2014). Biochemical and Biophysical Characterization of the Selenium-Binding and Reducing Site in Arabidopsis Thaliana Homologue to Mammals Selenium-Binding Protein 1. J. Biol. Chem..

[60] Stornaiuolo M., Lotti L.V., Borgese N., Torrisi M.-R., Mottola G., Martire G., Bonatti S. (2003). KDEL and KKXX Retrieval Signals Appended to the Same Reporter Protein Determine Different Trafficking between Endoplasmic Reticulum, Intermediate Compartment, and Golgi Complex. Mol. Biol. Cell.

[61] Hillier J., Allcott G.J., Guest L.A., Heaselgrave W., Tonks A., Conway M.E., Cherry A.L., Coles S.J. (2022). The BCAT1 CXXC Motif Provides Protection against ROS in Acute Myeloid Leukaemia Cells. Antioxidants.

[62] Kumar R.A., Koc A., Cerny R.L., Gladyshev V.N. (2002). Reaction Mechanism, Evolutionary Analysis, and Role of Zinc in Drosophila Methionine-R-Sulfoxide Reductase. J. Biol. Chem..

[63] Anelli T. (2002). ERp44, a Novel Endoplasmic Reticulum Folding Assistant of the Thioredoxin Family. EMBO J..

[64] Fomenko D.E., Gladyshev V.N. (2002). CxxS: Fold-independent Redox Motif Revealed by Genome-wide Searches for Thiol/Disulfide Oxidoreductase Function. Protein Sci..

[65] Fomenko D.E., Gladyshev V.N. (2003). Identity and Functions of CxxC-Derived Motifs. Biochemistry.

[66] Zhang L., Wang J.-C., Hou L., Cao P.-R., Wu L., Zhang Q.-S., Yang H.-Y., Zang Y., Ding J.-P., Li J. (2015). Functional Role of Histidine in the Conserved His-x-Asp Motif in the Catalytic Core of Protein Kinases. Sci. Rep..

[67] Yadav U., Rai T.K., Sethi S.C., Chandraker A., Khan M.A., Komath S.S. (2018). Characterising N-Acetylglucosaminylphosphatidylinositol de-N-Acetylase (CaGpi12), the Enzyme That Catalyses the Second Step of GPI Biosynthesis in Candida Albicans. FEMS Yeast Res..

[68] Zhang P., Zhang L., Hou Z., Lin H., Gao H., Zhang L. (2022). Structural Basis for the Substrate Recognition Mechanism of ATP-Sulfurylase Domain of Human PAPS Synthase 2. Biochem. Biophys. Res. Commun..

[69] Valassakis C., Livanos P., Minopetrou M., Haralampidis K., Roussis A. (2018). Promoter Analysis and Functional Implications of the Selenium Binding Protein (SBP) Gene Family in Arabidopsis Thaliana. J. Plant Physiol..

[70] Dutilleul C., Jourdain A., Bourguignon J., Hugouvieux V. (2008). The Arabidopsis Putative Selenium-Binding Protein Family: Expression Study and Characterization of SBP1 as a Potential New Player in Cadmium Detoxification Processes. Plant Physiol..

[71] Hugouvieux V., Dutilleul C., Jourdain A., Reynaud F., Lopez V., Bourguignon J. (2009). Arabidopsis Putative Selenium-Binding Protein1 Expression Is Tightly Linked to Cellular Sulfur Demand and Can Reduce Sensitivity to Stresses Requiring Glutathione for Tolerance. Plant Physiol..

[72] Fernandes A.P., Fladvad M., Berndt C., Andrésen C., Lillig C.H., Neubauer P., Sunnerhagen M., Holmgren A., Vlamis-Gardikas A. (2005). A Novel Monothiol Glutaredoxin (Grx4) from Escherichia Coli Can Serve as a Substrate for Thioredoxin Reductase. J. Biol. Chem..

[73] Meyer Y., Buchanan B.B., Vignols F., Reichheld J.-P. (2009). Thioredoxins and Glutaredoxins: Unifying Elements in Redox Biology. Annu. Rev. Genet..

[74] Rouhier N., Gelhaye E., Jacquot J.-P. (2004). Plant Glutaredoxins: Still Mysterious Reducing Systems. Cell. Mol. Life Sci. CMLS.

[75] Rouhier N., Lemaire S.D., Jacquot J.-P. (2008). The Role of Glutathione in Photosynthetic Organisms: Emerging Functions for Glutaredoxins and Glutathionylation. Annu. Rev. Plant Biol..

[76] Rey P., Becuwe N., Tourrette S., Rouhier N. (2017). Involvement of *Arabidopsis* Glutaredoxin S14 in the Maintenance of Chlorophyll Content. Plant Cell Environ..

[77] Cheng N.-H., Hirschi K.D. (2003). Cloning and Characterization of CXIP1, a Novel PICOT Domain-Containing Arabidopsis Protein That Associates with CAX1. J. Biol. Chem..

[78] Upchurch R.G. (2008). Fatty Acid Unsaturation, Mobilization, and Regulation in the Response of Plants to Stress. Biotechnol. Lett..

[79] Yang L., Ji J., Harris-Shultz K.R., Wang H., Wang H., Abd-Allah E.F., Luo Y., Hu X. (2016). The Dynamic Changes of the Plasma Membrane Proteins and the Protective Roles of Nitric Oxide in Rice Subjected to Heavy Metal Cadmium Stress. Front. Plant Sci..

[80] Chmielowska-Bąk J., Gzyl J., Rucińska-Sobkowiak R., Arasimowicz-Jelonek M., Deckert J. (2014). The New Insights into Cadmium Sensing. Front. Plant Sci..

[81] Maksymiec W., Wianowska D., Dawidowicz A.L., Radkiewicz S., Mardarowicz M., Krupa Z. (2005). The Level of Jasmonic Acid in Arabidopsis Thaliana and Phaseolus Coccineus Plants under Heavy Metal Stress. J. Plant Physiol..

[82] Maksymiec W. (2007). Signaling Responses in Plants to Heavy Metal Stress. Acta Physiol. Plant..

[83] Koeduka T., Matsui K., Hasegawa M., Akakabe Y., Kajiwara T. (2005). Rice Fatty Acid α-Dioxygenase Is Induced by Pathogen Attack and Heavy Metal Stress: Activation through Jasmonate Signaling. J. Plant Physiol..

[84] Mithöfer A., Schulze B., Boland W. (2004). Biotic and Heavy Metal Stress Response in Plants: Evidence for Common Signals. FEBS Lett..

[85] Maksymiec W. (2011). Effects of Jasmonate and Some Other Signalling Factors on Bean and Onion Growth during the Initial Phase of Cadmium Action. Biol. Plant..

[86] Swindell W.R. (2006). The Association Among Gene Expression Responses to Nine Abiotic Stress Treatments in Arabidopsis Thaliana. Genetics.

[87] Bernoux M., Timmers T., Jauneau A., Brière C., De Wit P.J.G.M., Marco Y., Deslandes L. (2008). RD19, an *Arabidopsis* Cysteine Protease Required for RRS1-R–Mediated Resistance, Is Relocalized to the Nucleus by the *Ralstonia solanacearum* PopP2 Effector. Plant Cell.

[88] Cheng Z., Guo X., Zhang J., Liu Y., Wang B., Li H., Lu H. (2020). βVPE Is Involved in Tapetal Degradation and Pollen Development by Activating Proprotease Maturation in Arabidopsis Thaliana. J. Exp. Bot..

[89] Köhnlein K., Urban N., Guerrero-Gómez D., Steinbrenner H., Urbánek P., Priebs J., Koch P., Kaether C., Miranda-Vizuete A., Klotz L.-O. (2020). A Caenorhabditis Elegans Ortholog of Human Selenium-Binding Protein 1 Is a pro-Aging Factor Protecting against Selenite Toxicity. Redox Biol..

